# A Rapid, Validated RP-HPLC Method for the Simultaneous Determination of Cleaning Validation and Cross-Contamination of 12 Beta-Lactam Compounds

**DOI:** 10.3797/scipharm.1208-20

**Published:** 2012-11-17

**Authors:** Harshal Kanubhai Trivedi, Nayan Kshtri, Mukesh C. Patel

**Affiliations:** 1 Analytical Research Lab, Cadila Pharmaceutical Ltd, Dholka-387 810, Gujarat, India.; 2 P. S. Science and H. D. Patel Arts College, S. V. Campus, Kadi-382 715, Gujarat, India.

**Keywords:** Chromatography, Cleaning validation, Cross contamination, Penicillin, Cephalosporin, HPLC

## Abstract

The present work reports a rapid reversed-phase high-performance liquid chromatography (RP-HPLC) method for the simultaneous determination of 12 beta-lactam components for cleaning validation and cross-contamination. A strategic experimental approach was implemented for the method development. The desired chromatographic separation was achieved on a Symmetry C18 (4.6 X 75 mm, 3.5 μm) column using gradient elution. The optimized mobile phase consisted of the buffer tetrabutylammonium hydroxide pH-6.8 and acetonitrile. The eluted compounds were monitored at 215 nm and 254 nm wavelength using a photodiode array detector. The developed method separated 12-beta-lactam compounds from each other within a run time of 50 min. The method is effective for the determination of cross-contamination of penicillin and cephalosporin production blocks. The present method is specific and a lower limit of quantification was determined on the basis of the signal-to-noise ratio method; it is 1 μg/mL for all components. The developed RP-HPLC method was validated according to the International Conference on Harmonization (ICH) guidelines.

## Introduction

The high allergic potential of drugs belonging to the penicillin family known as ß-lactam antibiotics makes the production and packaging of such products a focus of cross-contamination control in the pharmaceutical industry. These penicillin-derived compounds are used most often to treat a number of bacterial infections. The beta-lactam ring is common in both penicillin and the cephalosporin group.

Cleaning validation is a documented process that proves the effectiveness and consistency in cleaning pharmaceutical production equipment. Validations of equipment cleaning procedures are mainly used in pharmaceutical industries to prevent cross-contamination and adulteration of drug products, and hence, is critically important [1].The prime purpose of validating a cleaning process is to ensure compliance with federal and other standard regulations. The most important benefit of conducting such a validation work is the identification and correction of potential problems previously unsuspected, which could compromise the safety, efficacy, or quality of subsequent batches of drug products produced with the same equipment. The Unites States Food and Drug Administration (US FDA) guidelines for the manufacture of penicillin and cephalosporin antibiotics require such manufacture to be undertaken in dedicated facilities or on a campaign basis in a multiproduct facility. Campaign manufacture should only be undertaken after a thorough, fully-validated decontamination and cleaning operation between products. The FDA, if pressed, will also say that campaign manufacture is not encouraged and that it should not be considered as a long-term policy, but may be practical as a short-term expediency using closed systems, with a view to providing dedicated facilities in the longer term. In our experience, international regulatory authorities, including those of the European Union member states, have a similar policy for such and there is a considerable level of agreement on this issue. Penicillins and cephalosporins are, therefore, normally manufactured in separate dedicated facilities. It is unacceptable to manufacture both in the same facility for a further reason; since hypersensitivity reactions to penicillins are not necessarily seen in the same patient population as for cephalosporins, it is quite common for people who are hypersensitive to penicillins to be prescribed cephalosporins. Consequently, trace contamination of one product type by the other is highly undesirable. A dedicated facility can be very costly, representing a significant investment even for a large company. When the demand for the antibiotic falls, the facility may be needed by the company for another purpose. At this stage, it is necessary to clean and decontaminate the facility prior to taking it into an alternative usage [2].

Marie *et al.* reported a RP-HPLC method for the simultaneous determination of 12 beta-lactam antibiotics in human plasma [3]. Ni YN et al. reported a simultaneous spectrophotometric method for the determination of certain beta-lactam antibiotics in rabbit serum using multivariate calibration [4]. Fukutsu et al. reported a RPLC-MS method for the verification of cefmetazole and cefpodoxime proxetil contamination in other pharmaceuticals [5]. Takeba et al. reported the iron-pair liquid chromatography method for the determination of beta-lactam antibiotics in milk [6]. Also, some capillary electrophoresis methods are available for the determination of beta-lactam compounds [7, 8]. Some liquid chromatography methods are available for the determination of beta-lactam in serum, body fluids, and drugs [9–14]. Some LC-MS methods are also available for the determination of beta-lactam compounds [15, 16]. Not a single analytical method is available for the simultaneous determination of the 12 beta-lactam compounds for cleaning validation and cross-contamination.

The aim of the present work was the development of a RP-HPLC method for the determination of 12 beta-lactam compounds simultaneously. Therefore, the RP-HPLC method was developed for the simultaneous determination of 12 beta-lactam compounds. The developed method was validated according to International Conference on Harmonization (ICH) guidelines [17] to show the capability of the method.

## Results and Discussion

### Method development and optimization

The main criteria for development of a RP-HPLC method for the simultaneous determination of 12 beta-lactam compounds during a cleaning validation and cross-contamination study are as follows: the method should be able to quantify all 12 beta-lactam compounds in a single run and should be accurate, reproducible, linear, free of interference from blank and swab, and enough for the routine use in quality control laboratories.

### Analytical parameters and validation

After satisfactory development of the method, it was subjected to method validation as per ICH guidelines [17]. The method was validated to demonstrate that it is suitable for its intended purpose by the standard procedure to evaluate adequate validation characteristics (specificity, system suitability, accuracy, linearity, and limit of quantification).

### Specificity

**Fig. 2:**
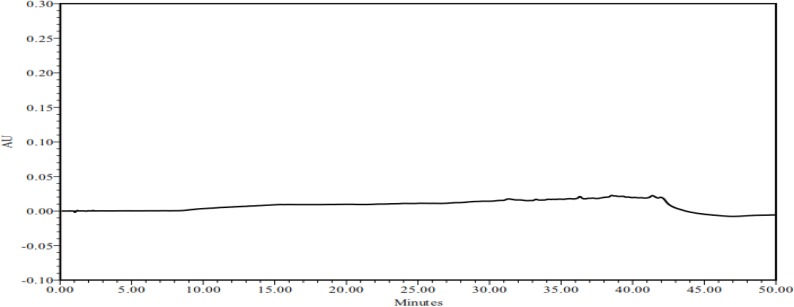
Specimen chromatogram of blank

**Fig. 3:**
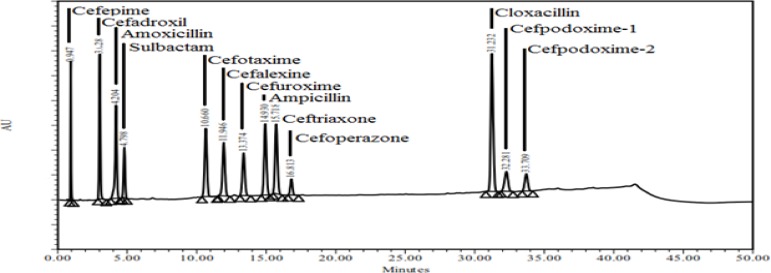
Specimen chromatogram of composite beta-lactam standard

Specificity is the ability of the method to measure the analyte response in the presence of diluent. Figure 2 and 3 show that there is no interference at the RT (retention time) for all 12 beta-lactam compounds due to the blank.

### Precision

#### Instrument precision: (Suitability of system)

System suitability parameters were measured so as to verify the system performance. System precision was determined by six replicate injections of standard preparation. All important characteristics including % RSD, resolution (between all nearest peaks), tailing factor, and theoretical plate number were measured. The % RSD of area counts of six replicate injections for all beta-lactam peaks were below 2.0 %, and the resolution between the two nearest peaks was more than 1.5. These indicate that the system is precise and suitable for determination of cleaning validation and cross-contamination of all 12 beta-lactam compounds. The results obtained are shown in Table 3. The parameters all complied with the acceptance criteria and system suitability was established.

### Accuracy

The accuracy of an analytical method is the closeness of test results obtained by that method compared with the true values. To confirm the accuracy of the proposed method, recovery experiments were carried out by the standard addition technique. The accuracy of the method was carried out by adding known amounts of each drug corresponding to three concentration levels; 50, 100, and 150% of the target concentration on 2X2 inch SS-316 plates in triplicate. The samples were given the same treatment as described as per cleaning procedure of validation. The percentage recoveries of all components at each level and each replicate were determined. The mean of percentage recoveries (n=3) and the relative standard deviation were calculated. The amount recovered was within ± 15.0 % of the amount added, which indicates that the method is suitable for the determination of cross-contamination and cleaning validation study. It was confirmed from results that the method is accurate (Table 4).

### Linearity

**Fig. 4:**
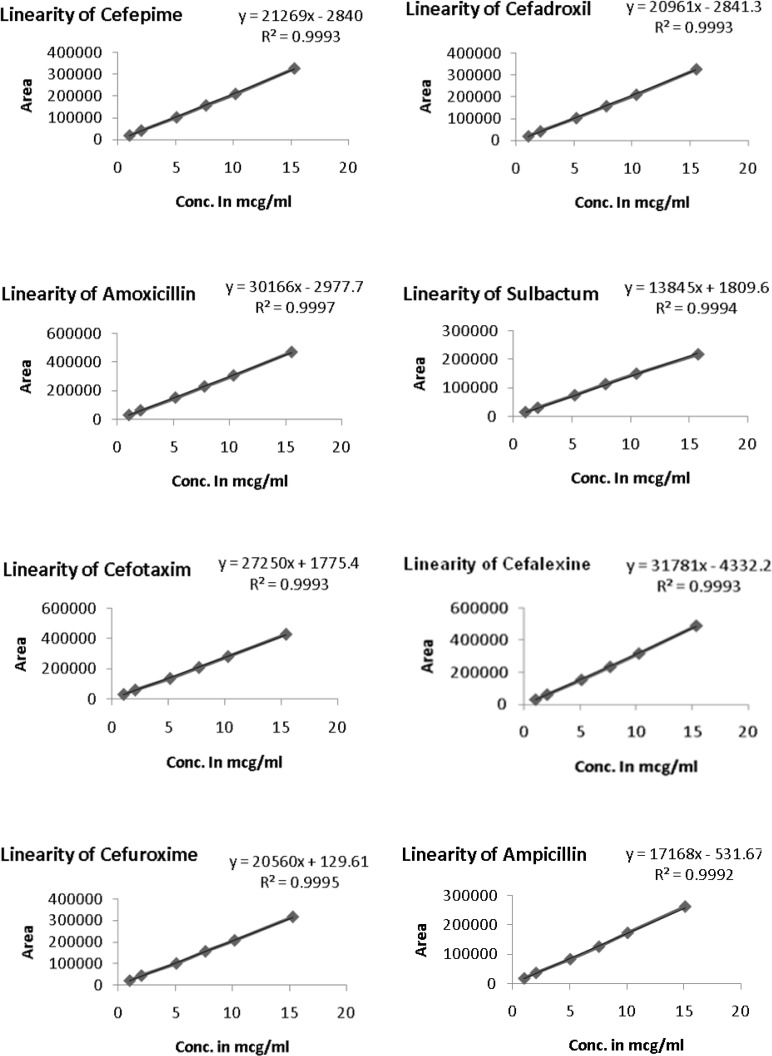

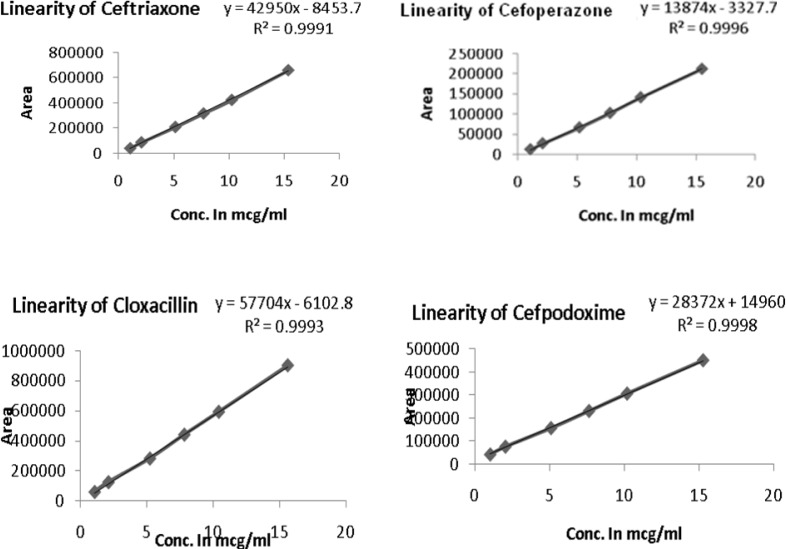
Linearity curve of Beta-Lactam compounds

The linearity of an analytical method is its ability to elicit test results that are directly, or by a well-defined mathematical transformation, proportional to the concentration of the analyte in a sample within a given range. The nominal (100%) concentrations of standard and test solutions for all components were 5 μg/mL. The response function was determined by preparing standard solutions at six different concentration levels ranging from 1–15 μg/mL for all components (LOQ to 300 % of the analyte concentration). The response was found to be linear from the LOQ (1 μg/mL) to 300% (15 μg/mL) of the standard concentration. For all the compounds, the correlation coefficients were greater than 0.999. The linearity concentration and the regression statistics are shown in Tables 5 and 6 respectively. The linearity curves for all components are presented in Figure 4.

### Limit of Quantification (LOQ)

**Fig. 5:**
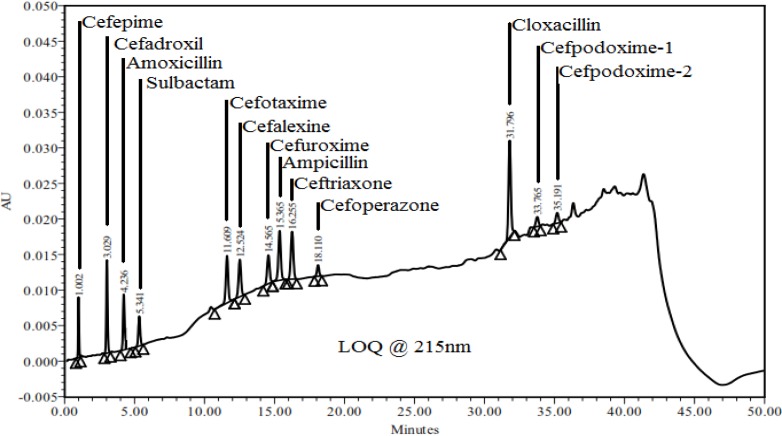
Chromatogram of LOQ at 215 nm

**Fig. 6:**
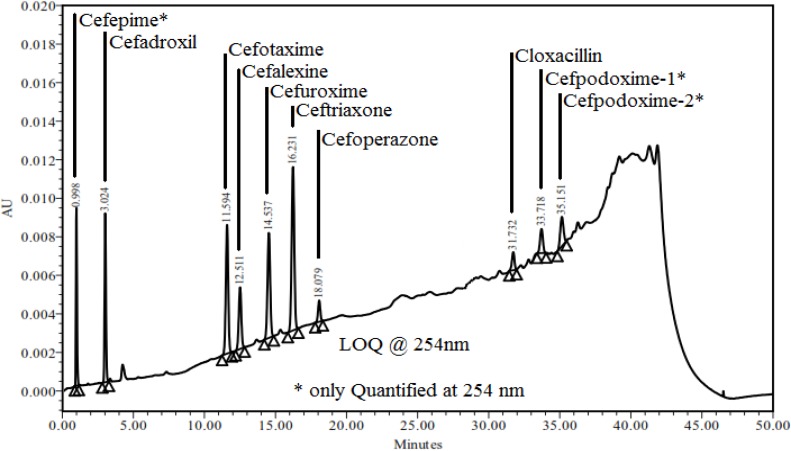
Chromatogram of LOQ at 254 nm

The signal-to-noise ratio (*S/N*) method was adopted for the determination of the lower limit of quantification. The limit of quantification is estimated to be ten times the *S/N* ratio. The quantification limit was achieved by injecting a series of possible dilute solutions of all components and the precision was established at the quantification level. The % RSD of peak areas was well within the acceptance limit, not more than 10%. The determined lower limit of qualification and precision at LOQ values for all components are presented in Table 7. LOQ chromatograms are presented in Figures 5 and 6.

## Experimental

### Materials and Reagents

All active pharmaceutical ingredients were provided by Cadila pharmaceutical Ltd., Ahmedabad, India. All working standards were also provided by Cadila pharmaceutical Ltd., Ahmedabad, India. HPLC grade acetonitrile, tetrabutylammonium hydroxide, and ortho phosphoric acid were obtained from Spectrochem Ltd. The 0.22 μm PVDF membrane filter was purchased from Pall Life Science Limited (India). The 0.22 μm PVDF syringe filter was purchased from Millipore (India). High purity water was generated by using the Milli-Q Plus water purification system (Millipore, Milford, MA, USA).

### Chromatographic conditions

Analyses were performed on the Waters Alliance 2487 system (Waters, Milford, USA), consisting of a binary solvent manager, sample manager, and PDA (photodiode array) detector. System control, data collection, and data processing were accomplished using Waters Empower^™^-2 chromatography data software. The chromatographic condition was optimized using the Waters Symmetry C18, 3.5 μm (75 mm x 4.61 mm) column. A mixture of a buffer (pH-6.8) and acetonitrile was used as solvent A in the ratio of (850:150) and solvent B in the ratio of (250:750). We transferred 13.2 ml of 40% aqueous solution of tetrabutylammonium hydroxide to 4000 ml Milli-Q water then adjusted the pH-6.8 with ortho phosphoric acid. This was then filtered through a 0.22 μm PVDF membrane filter and degassed under vacuum prior to use. The separation of all components was achieved by gradient elution using solvent A and B (Table 8). Solution A was used as diluent. The final selected and optimized conditions were as follows; injection volume 20 μL, gradient elution (Table 8), at a flow rate of 1.0 mL/min at 35°C (column oven) temperature, detection wavelength 215 and 254 nm, and sample temperature 15°C. Under these conditions, the backpressure in the system was about 2500 psi.

### Standard solution preparation

The standard solution was prepared by dissolving standard substances in diluent to obtain solutions containing 5 μg/mL of each component.

### Sample solution preparation

The swab was transferred into the test tube and 10 ml of diluent was added, then the test tube was sonicated for 10 minutes with shaking. This solution was filtered with a 0.22μ PVDF syringe filter.

## Conclusion

A gradient RP-HPLC method was successfully developed for the estimation of 12 beta-lactam components for cleaning validation and cross-contamination in the pharmaceutical production area. The method validation results proved that the method is selective, accurate, and linear. Moreover, it may be applied for the determination of cross-contamination and cleaning validation of beta-lactam components.

## Figures and Tables

**Fig. 1:**
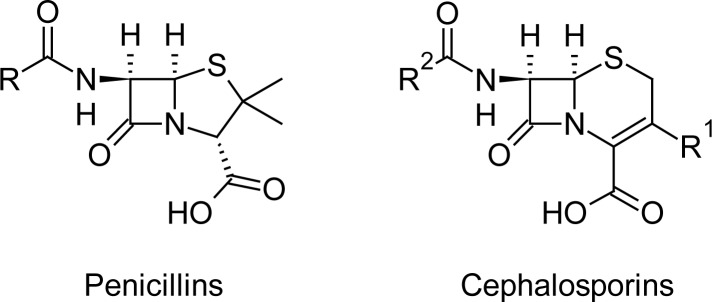
Core structure of penicillins and cephalosporins

**Tab. 1.** Chemical structure and IUPAC name of all analyzed beta-lactam compounds.

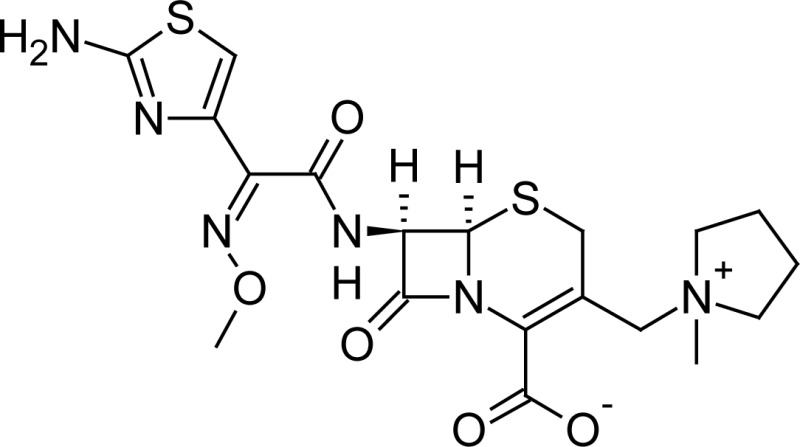 Cefepime (6*R*,7*R*)-7-{[(2*Z*)-2-(2-amino-1,3-thiazol-4-yl)-2-(methoxyimino)acetyl]amino}-3-[(1-methyl-pyrrolidinium-1-yl)methyl]-8-oxo-5-thia-1-azabicyclo[4.2.0]oct-2-ene-2-carboxylate	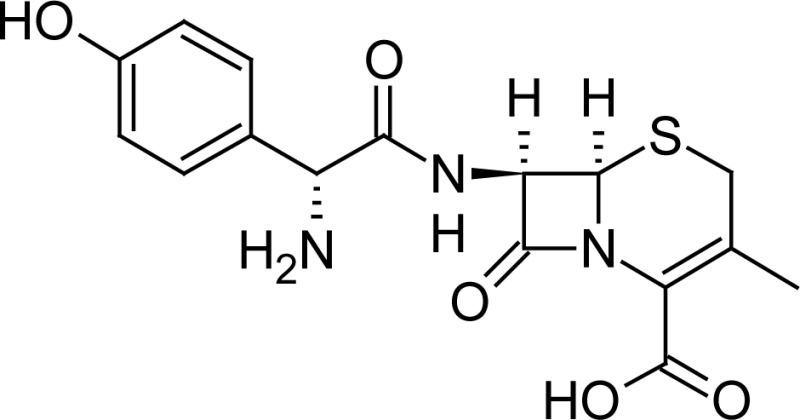 Cefadroxil (6*R*,7*R*)-7-{[(2*R*)-2-amino-2-(4-hydroxyphenyl)-acetyl]amino}-3-methyl-8-oxo-5-thia-1-azabicyclo[4.2.0]oct-2-ene-2-carboxylic acid
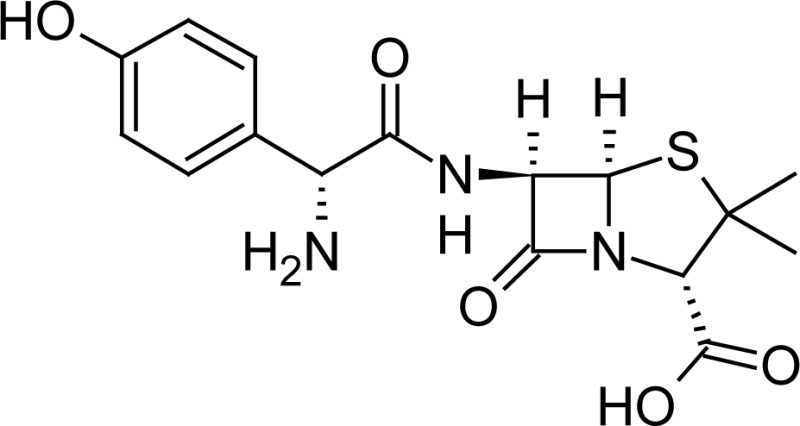 Amoxicillin (2*S*,5*R*,6*R*)-6-{[(2*R*)-2-amino-2-(4-hydroxyphenyl)-acetyl]amino}-3,3-dimethyl-7-oxo-4-thia-1-azabicyclo[3.2.0]heptane-2-carboxylic acid	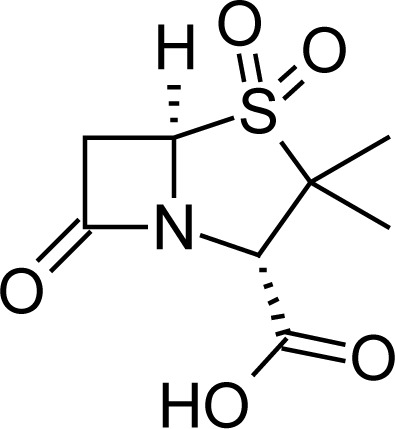 Sulbactam (2*S*,5*R*)-3,3-dimethyl-7-oxo-4-thia-1-aza-bicyclo[3.2.0]heptane-2-carboxylic acid 4,4-dioxide
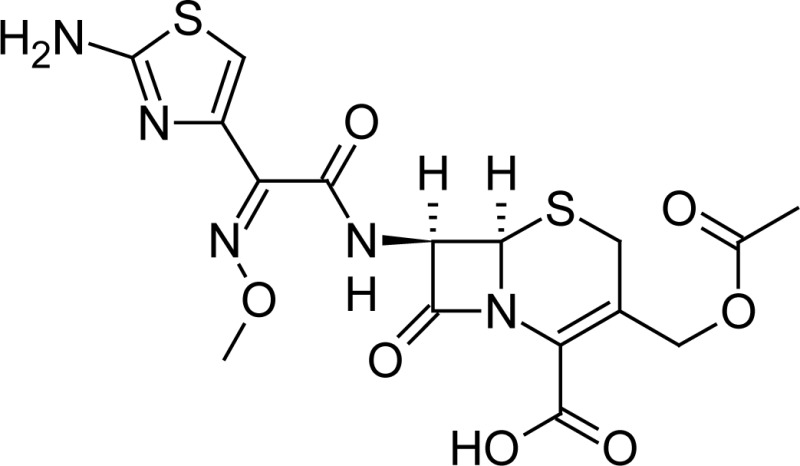 Cefotaxime (6*R*,7*R*)-3-[(acetyloxy)methyl]-7-{[(2 *Z*)-2-(2-amino-1,3-thiazol-4-yl)-2-(methoxyimino)acetyl]amino}-8-oxo-5-thia-1-azabicyclo[4.2.0]oct-2-ene-2-carboxylic acid	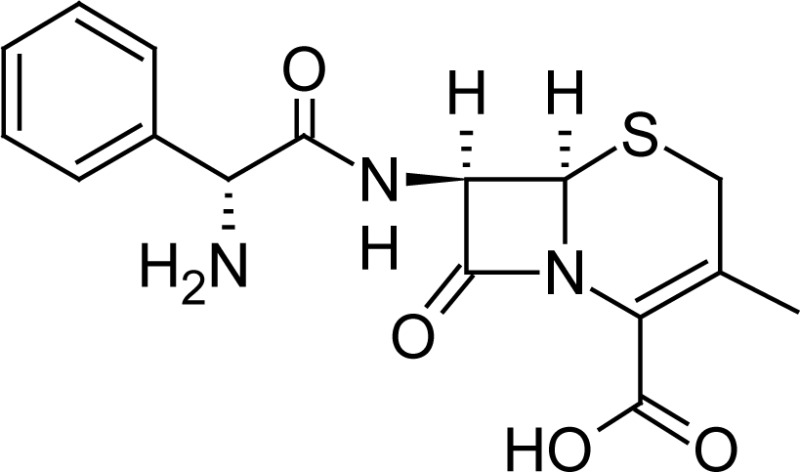 Cefalexin (6*R*,7*R*)-7-{[(2*R*)-2-amino-2-phenylacetyl]amino}-3-methyl-8-oxo-5-thia-1-aza-bicyclo[4.2.0]oct-2-ene-2-carboxylic acid
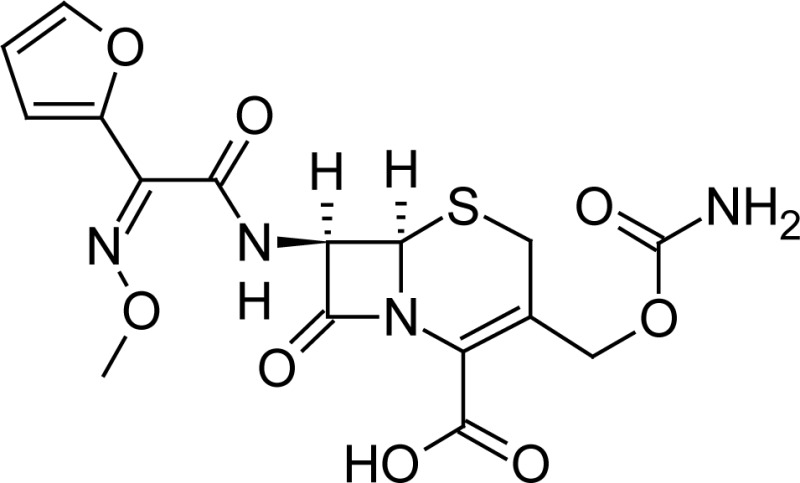 Cefuroxime (6*R*,7*R*)-3-[(carbamoyloxy)methyl]-7-{[(2*Z*)-2-(furan-2-yl)-2-(methoxyimino)acetyl]amino}-8-oxo-5-thia-1-azabicyclo[4.2.0]oct-2-ene-2-carboxylic acid	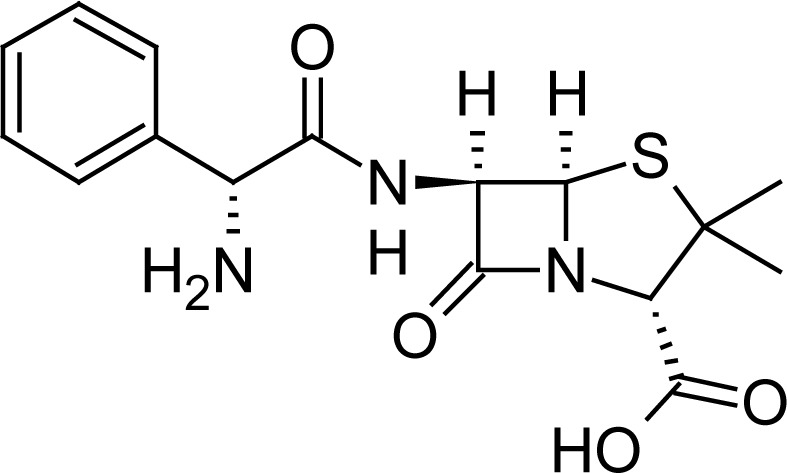 Ampicillin (2*S*,5*R*,6*R*)-6-{[(2 *R*)-2-amino-2-phenyl-acetyl]amino}-3,3-dimethyl-7-oxo-4-thia-1-azabicyclo[3.2.0]heptane-2-carboxylic acid
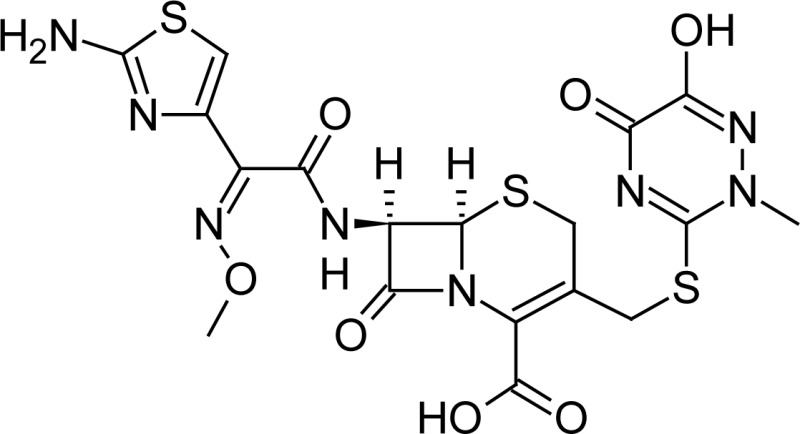 Ceftriaxone (6*R*,7*R*)-7-{[(2*Z*)-2-(2-amino-1,3-thiazol-4-yl)-2-(methoxyimino)acetyl]amino}-3-{[(6-hydroxy-2-methyl-5-oxo-2,5-dihydro-1,2,4-triazin-3-yl)-sulfanyl]methyl}-8-oxo-5-thia-1-azabicyclo[4.2.0]oct-2-ene-2-carboxylic acid	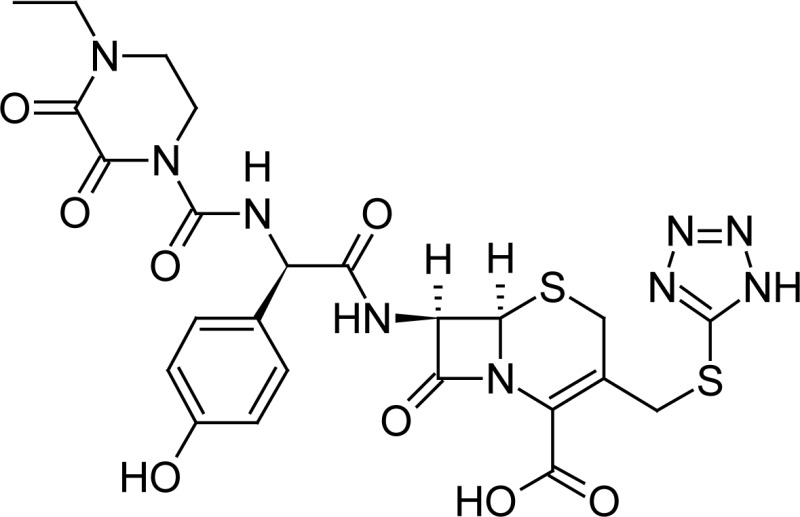 Cefoperazone (6*R*,7*R*)-7-{[(2*R*)-2-{[(4-ethyl-2,3-dioxopiperazin-1-yl)-carbonyl]amino}-2-(4-hydroxyphenyl)acetyl]amino}-8-oxo-3-[(1*H* -tetrazol-5-ylsulfanyl)methyl]-5-thia-1-azabicyclo[4.2.0]oct-2-ene-2-carboxylic acid
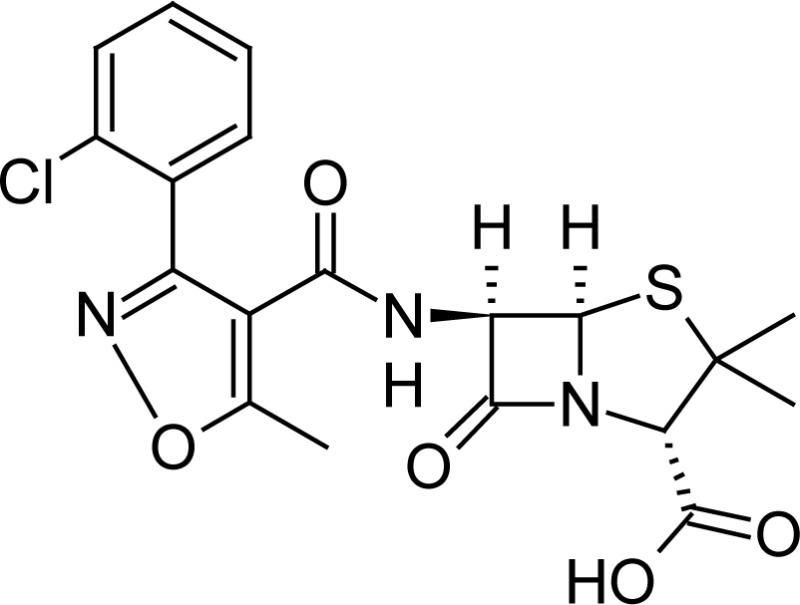 Cloxacillin (2*S*,5*R*,6*R*)-6-({[3-(2-chlorophenyl)-5-methylisoxazol-4-yl]carbonyl}amino)-3,3-dimethyl-7-oxo-4-thia-1-azabicyclo[3.2.0]heptane-2-carboxylic acid	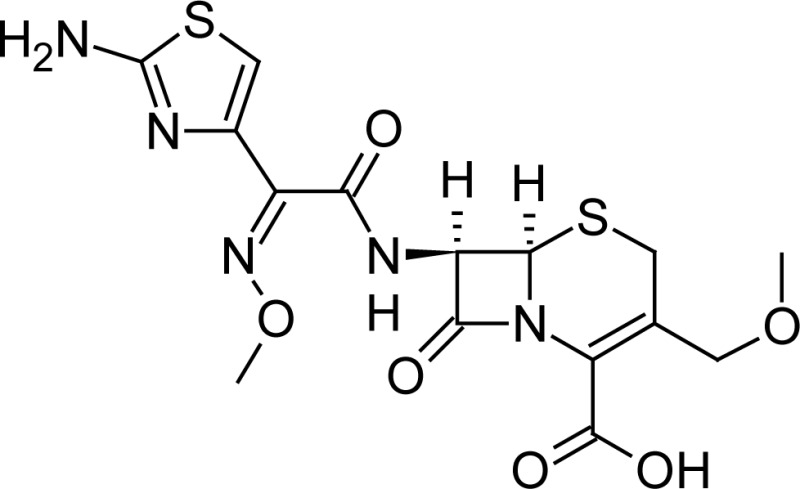 Cefpodoxime (6*R* ,7*R*)-7-{[(2*Z*)-2-(2-amino-1,3-thiazol-4-yl)-2-(methoxyimino)acetyl]amino}-3-(methoxymethyl)-8-oxo-5-thia-1-azabicyclo[4.2.0]oct-2-ene-2-carboxylic acid

**Tab. 2.** Summary of peak purity results

**Name of compound**	**Retention time**	**Resolution between nearest peaks**	**Purity flag**
Cefepime Hydrochloride	0.95	Not applicable	Pass
Cefadroxil Monohydrate	3.03	15.45	Pass
Amoxicillin Trihydrate	4.20	6.18	Pass
Sulbactam Sodium	4.79	5.96	Pass
Cefotaxime Sodium	10.66	21.55	Pass
Cefalexine Monohydrate	11.95	2.13	Pass
Cefuroxime Sodium	13.38	6.87	Pass
Ampicillin Trihydrate	14.93	1.74	Pass
Ceftriaxone Sodium	15.72	3.60	Pass
Cefoperazone Sodium	16.81	5.56	Pass
Cloxacillin Sodium	31.23	45.07	Pass
CefpodoximeProxetil-I	32.28	4.62	Pass
CefpodoximeProxetil-II	33.70	3.65	Pass

**Tab. 3.** Summary of system suitability parameter

**Name of compound**	**Theoretical plates**	**Tailing factor**	**Resolution between nearest peak**	**% RSD**
Cefepime	2154	1.1	Not applicable	0.56
Cefadroxil	2236	1.1	15.45	0.50
Amoxicillin	5642	1.2	6.18	0.31
Sulbactam	6782	1.3	5.96	0.69
Cefotaxime	7823	1.1	21.55	0.47
Cefalexine	9483	1.2	2.13	1.34
Cefuroxime	10521	1.6	6.87	1.64
Ampicillin	10215	1.2	1.74	1.12
Ceftriaxone	11546	1.3	3.60	0.55
Cefoperazone	10124	1.1	5.56	0.95
Cloxacillin	11269	1.2	45.07	0.72
Cefpodoxime-I	11456	1.3	4.62	0.96
Cefpodoxime-II	12546	1.2	3.65	0.85

**Tab. 4.** Accuracy results

**Substance**		**At 50% (2.5** μg/mL**)**	**At 100% (5** μg/mL**)**	**At 150% (10** μg/mL**)**
Cefepime	% Recovery^#^	87.2	92.1	99.3
	% R.S.D.*	3.5	4.2	2.7

Cefadroxil	% Recovery^#^	91.8	94.1	93.4
	% R.S.D.*	3.2	2.2	3.7

Amoxicillin	% Recovery^#^	86.1	89.1	91.2
	% R.S.D.*	4.3	3.6	2.7

Sulbactam	% Recovery^#^	88.9	87.5	92.3
	% R.S.D.*	3.9	4.1	3.5

Cefotaxime	% Recovery^#^	91.8	97.6	101.4
	% R.S.D.*	4.1	2.7	3.1

Cefalexine	% Recovery^#^	91.2	95.9	96.1
	% R.S.D.*	2.7	3.9	3.2

Cefuroxime	% Recovery^#^	87.3	89.2	90.5
	% R.S.D.*	4.7	4.1	3.5

Ampicillin	% Recovery^#^	88.3	98.4	97.3
	% R.S.D.*	4.2	4.3	3.1

Ceftriaxone	% Recovery^#^	99.3	101.4	102.3
	% R.S.D.*	3.1	2.2	2.8

Cefoperazone	% Recovery^#^	95.1	92.3	96.7
	% R.S.D.*	3.8	3.1	2.7

Cloxacillin	% Recovery^#^	97.7	98.1	99.6
	% R.S.D.*	2.9	3.3	3.6

Cefpodoxime	% Recovery^#^	98.3	99.1	95.7
	% R.S.D.*	3.5	4.1	3.8

* Determined on three values;

# Mean of three determinations.

**Tab. 5.** Linearity Concentration

**Sr. No**	**Sample ID**	**Concentration in μg/mL**
1	LOQ Solution	1
2	50% Linearity Level	2.5
3	100% Linearity Level	5
4	150% Linearity Level	7.5
5	200% Linearity Level	10
6	300% Linearity Level	15

**Tab. 6.** Regression statistics

**Compound**	**Linearity range (μg/mL)**	**Correlation Coefficient (r^2^)**	**Linearity (Equation)**
Cefepime	1.02–15.30	0.999	y = 21269x – 2840
Cefadroxil	1.04–15.53	0.999	y = 20961x – 2841
Amoxicillin	1.04–15.53	0.999	y = 30166x – 2977
Sulbactam	1.05–15.75	0.999	y = 13845x + 1809
Cefotaxime	1.03–15.45	0.999	y = 27250x + 1775
Cefalexine	1.03–15.38	0.999	y = 31781x – 4332
Cefuroxime	1.02–15.30	0.999	y = 20560x + 129.6
Ampicillin	1.01–15.11	0.999	y = 17168x – 531.6
Ceftriaxone	1.03–15.38	0.999	y = 42950x – 8453
Cefoperazone	1.04–15.53	0.999	y = 13874x – 3327
Cloxacillin	1.04–15.60	0.999	y = 57704x – 6102
Cefpodoxime	1.02–15.30	0.999	y = 28372x + 14960

**Tab. 7.** LOQ and LOQ precision

**Name of component**	**LOQ (μg/mL)**	Precision (% RSD*)
Cefepime	1.616	0.50
Cefadroxil	2.286	0.76
Amoxicillin	1.097	0.93
Sulbactam	1.550	1.77
Cefotaxime	1.490	1.73
Cefalexine	1.770	1.68
Cefuroxime	1.341	1.89
Ampicillin	1.880	1.93
Ceftriaxone	1.790	1.14
Cefoperazone	1.262	1.80
Cloxacillin	1.136	1.82
Cefpodoxime	0.994	1.22

* Determined on six values

**Tab. 8.** Gradient elution

**Time in min**	**Solvent-A**	**Solvent-B**
0	100	0
6	100	0
18	85	15
40	60	40
45	100	0
50	100	0
